# CCR5 and Biological Complexity: The Need for Data Integration and Educational Materials to Address Genetic/Biological Reductionism at the Interface of Ethical, Legal, and Social Implications

**DOI:** 10.3389/fimmu.2021.790041

**Published:** 2021-12-02

**Authors:** Jacob Bauss, Michele Morris, Rama Shankar, Rosemary Olivero, Leah N. Buck, Cynthia L. Stenger, David Hinds, Joshua Mills, Alexandra Eby, Joseph W. Zagorski, Caitlin Smith, Sara Cline, Nicholas L. Hartog, Bin Chen, John Huss, Joseph A. Carcillo, Surender Rajasekaran, Caleb P. Bupp, Jeremy W. Prokop

**Affiliations:** ^1^ Department of Pediatrics and Human Development, College of Human Medicine, Michigan State University, Grand Rapids, MI, United States; ^2^ HudsonAlpha Institute for Biotechnology, Huntsville, AL, United States; ^3^ Infectious Disease, Helen DeVos Children’s Hospital, Grand Rapids, MI, United States; ^4^ Department of Mathematics, University of North Alabama, Florence, AL, United States; ^5^ Department of Biology, Grand Valley State University, Allendale, MI, United States; ^6^ Department of Science, Davenport University, Grand Rapids, MI, United States; ^7^ Office of Research, Spectrum Health, Grand Rapids, MI, United States; ^8^ Department of Biology, Athens State University, Athens, AL, United States; ^9^ Allergy & Immunology, Spectrum Health, Grand Rapids, MI, United States; ^10^ Department of Pharmacology and Toxicology, Michigan State University, East Lansing, MI, United States; ^11^ Department of Philosophy, The University of Akron, Akron, OH, United States; ^12^ Department of Critical Care Medicine and Pediatrics, Children’s Hospital of Pittsburgh, University of Pittsburgh School of Medicine, Pittsburgh, PA, United States; ^13^ Pediatric Intensive Care Unit, Helen DeVos Children’s Hospital, Grand Rapids, MI, United States; ^14^ Medical Genetics, Spectrum Health, Grand Rapids, MI, United States

**Keywords:** CCR5, viral infections, expression analysis, evolutionary profiling, molecular dynamic simulations, microglia, educational material generation

## Abstract

In the age of genomics, public understanding of complex scientific knowledge is critical. To combat reductionistic views, it is necessary to generate and organize educational material and data that keep pace with advances in genomics. The view that CCR5 is solely the receptor for HIV gave rise to demand to remove the gene in patients to create host HIV resistance, underestimating the broader roles and complex genetic inheritance of CCR5. A program aimed at providing research projects to undergraduates, known as CODE, has been expanded to build educational material for genes such as *CCR5* in a rapid approach, exposing students and trainees to large bioinformatics databases and previous experiments for broader data to challenge commitment to biological reductionism. Our students organize expression databases, query environmental responses, assess genetic factors, generate protein models/dynamics, and profile evolutionary insights into a protein such as CCR5. The knowledgebase generated in the initiative opens the door for public educational information and tools (molecular videos, 3D printed models, and handouts), classroom materials, and strategy for future genetic ideas that can be distributed in formal, semiformal, and informal educational environments. This work highlights that many factors are missing from the reductionist view of CCR5, including the role of missense variants or expression of CCR5 with neurological phenotypes and the role of CCR5 and the delta32 variant in complex critical care patients with sepsis. When connected to genomic stories in the news, these tools offer critically needed Ethical, Legal, and Social Implication (ELSI) education to combat biological reductionism.

## Introduction

Genetics and genomics are complex. Nearly every scientist is trained to integrate the scientific method into research design, formulating a hypothesis and testing it. However, this method of probing scientific insights was formulated in an age with limited data and resources in a simplified, often reductionistic, biological understanding. As the amount of data generated now often overcomes what a mind can comprehend, hypothesis-driven research becomes more and more challenging, especially when clinical, real-world decision-making occurs. Focused, hypothesis-driven research in genomics can often result in overly simplified views of genes that result in reductionism when not balanced with a full view of the biological complexity. To combat these reductionistic views in genomics, it is critical to look more broadly, often non-hypothesis driven and based on the larger data analysis. It is the responsibility of the genetics community to build tools that combat misunderstanding and reductionism (1), particularly when Ethical, Legal and Social Implications (ELSI) are involved. The big data community often is embraced to move beyond gene to single-function insights to broaden our view of how genetics contributes to biology.

Throughout our educational pipeline, genomic literacy has been a growing weakness, even in well-educated individuals, potentially elevating genetic essentialism (2, 3). We cannot utilize only scientific publications to combat genomic reductionism, as these are not accessible to most individuals. Studies in high school standards (4), undergraduate education (5), medical school training (6), specialized medical fields such as nursing (7, 8), practicing physicians (9), and general public education (10, 11) all suggest weaknesses in our genomics education pipelines. Many of our genomics classes, textbooks, and resources still focus on reductionistic genetics of Punnett squares and monogenic inheritance, missing the complexity of genomics (12). In a randomized control trial, it has been shown that students with more genomic literacy prevents essentialist views of genetics (13). The Public Understanding and Attitudes towards Genetics and Genomics (PUGGS) instrument applied to first-year university students suggested that the challenges of genetic reductionism also include social factors of age and religion (14). However, a more recent assessment of the PUGGS suggests the need for reform and further applications to educational assessments (15). Through a mixed-methods approach, others have shown the need to implement culturally and linguistically diverse backgrounds into our genomics education (16), which could be accomplished with more visual aids and interactive forums. The increasing education on gene regulation, epigenetics, and gene-by-environment regulation is critically needed at earlier levels to counter reductionistic views (17). Implementing more mechanistic reasoning abilities into genomic literacy is also critical (18). Here we lay out a strategy to engage students in complex research on genes, integrating large data resources into educational tools that others can be used to broaden genomics perspectives.

The fundamental insight of CCR5 as a receptor for HIV to infect cells, and that a human variant known as delta32 (hg38 3_46373452_TACAGTCAGTATCAATTCTGGAAGAATTTCCAG_T, rs333/rs775750898, CCR5 p.Ser185IlefsTer32) in the protein corresponds to HIV resistance (19, 20), gave rise to the potential to target *CCR5* for HIV treatment and prophylaxis (21–23). What started as a potential to edit blood cells to give host HIV resistance (24, 25) created ambiguity and opportunities for scientists to perform the gene editing in human germline experiments. Yet, these germline experiments leave out many of the risks of the experiments ranging from off-target activity (26) to the role *CCR5* plays in normal cell, tissue, and organ biology (27), which can be compensated by the complex multivariant inheritance of delta32. While the potential for CRISPR editing of *CCR5* to create HIV resistance is intriguing (28), a more complex understanding of *CCR5* biology is critical. HudsonAlpha Institute for Biotechnology and Michigan State University formulated the Characterizing Our DNA Exceptions (CODE) program to advance knowledge of genetic variation and provide insights into genetics through a research program for undergraduate and graduate students in performing gene-centric data surveillance and integration into knowledge. This program created an opportunity for students within our CODE program to build CCR5 tools for educational use to broaden understanding of the biology of CCR5. This work describes the tools and resources integrated for a richer, more complex view of CCR5, with tools and resources accessible outside of our traditional publication system that does not often reach those needing enhanced genomic literacy.

The C-C Chemokine Receptor Type 5 (CCR5) is a G-protein coupled receptor (GPCR) prominently known for its role as the co-receptor (with CD4 as the primary receptor) in HIV infection. However, this receptor has many roles outside of the infectious disease realm. CCR5 is predominantly located on the cell membrane of macrophages, T-cells, Hofbauer cells, and Kupffer cells with minor expression on epithelial cells, type 2 alveolar cells, fibroblasts, and B-cells (29). When comparing *CCR5* expression among T cell subpopulations, it was found to be specific to TH1 T-cells (30) and CD8+ T-cells (31) as opposed to the TH2 subpopulation involved in allergy and parasitic responses, which were more specific for CCR3 (32). Ellwanger et al. have laid out many of the pros and cons of CCR5 removal, including a detailed literature review of the many experiments performed for CCR5 biology outside of HIV (33). In 2009, it was well laid out that the recent emergence of the HIV infection could not be a sole explanation for the emergence of delta32, instead suggesting a push-pull aspect of immune activation, where inhibition of immune overactivation due to infection or autoimmunity could be evolutionarily advantageous, but with consequences to immune system components (34). This is further defended by data in CCR5 knockout models, which suggest advantageous roles in decreased immune activation (35–37) while having neurological complications (38, 39) and, in some cases, blunted immune response to pathogens (40, 41). As CCR5 has also been extensively linked to autoimmunity and autoimmune liver diseases, targeting it with therapeutics has been suggested (42–44).

CCR5 is a receptor for several CC-chemokines, including CCL3 (MIP-1-alpha), CCL4 (MIP-1-beta), and CCL5 (RANTES), which induce intracellular signal amplification *via* activation of the AKT and NF-KB pathways (45, 46). Both CCL3 and CCL4 are predominantly produced and secreted by T-cells, Hofbauer cells, macrophages, and Kupffer cells, while CCL5 has higher expression and secretion by T-cells (29). When bound to CCR5, CCL3 plays a significant role in T-cell chemotaxis and transmigration with similar activities in macrophages and other immune cells (47, 48). CCL4 is a potent chemotactic factor for neutrophils (49), with knock-out studies demonstrating decreased neutrophil chemotaxis to sites of inflammation (50). CCL5 plays a role in the cellular migration of T-cells, NK cells, macrophages, eosinophils, and basophils (51). CCL5 production has also been shown to reduce HIV entry into host cells (52). Homology within the C-C Chemokine Receptor family may compensate for some of the CCR5 biology, but the extent to which these mechanisms can compensate for the broad phenotypes of CCR5 ligand activation within individuals carrying the delta32 or other CCR5 variants is not well understood. Therefore, we have integrated our CCR5 knowledgebase with that of the larger C-C Chemokine Receptor family and broader phenotypic knowledge, using publicly available data, to expand our understanding of CCR5, which is critical in establishing a broader biological context for understanding the consequences of genetic manipulation. This example demonstrates how public data needs to be better integrated before setting out on high-risk clinical experiments.

## Methods

### Amino Acid Knowledgebase and Human Genomic Variants

The human CCR5 protein sequence (UniProt P51681) was assessed on NCBI BLAST (53) against the *Homo sapiens* UniProtKB/Swiss-Prot database, and the top 100 hits were extracted for the canonical UniProt isoform. These 100 sequences were aligned using ClustalW (54), alignment available at https://doi.org/10.6084/m9.figshare.16619983, and a phylogenetic tree (https://doi.org/10.6084/m9.figshare.16619950) was constructed using MEGA (55) with 500 bootstrap calculations. Amino acids of the alignment were exported into Excel, where the conservation to all 100 GPCR sequences was calculated for each amino acid of human CCR5. The conservation was also calculated for the top 16 BLAST hits with an E-value less than 1E-50 (CCR5, CCR2, CCR1, CCR4, CCR3, CCR8, CCRL2, CCR9, CX3CR1, CCR6, XCR1, CCR7, CXCR4, ACKR2, ACKR4, CXCR6). Vertebrate orthologs of *CCR5* were extracted from NCBI ortholog as RefSeq transcripts, which were parsed for open reading frames using TransDecoder-v5.5 (56) and aligned using ClustalW codon (alignment available at https://doi.org/10.6084/m9.figshare.16619986). The translated amino acid sequences were assessed for percent conservation relative to the human CCR5 sequence or were assessed for functional conservation based on hydrophobic (A, V, I, L, M, F, Y, W), aromatic (F, Y, W, H), polar basic (R, H, K), polar acidic (D, E), or Ser/Thr (S, T) amino acids. Codon selection and linear motif analysis of the open reading frame alignment were calculated as previously described (57, 58). Knowledge for human CCR5 topology, modifications, mutagenesis, and natural variants were extracted from the UniProt database (59) on 6/15/2021.

The human CCR5 (UniProt P51681) protein was modeled using homology modeling in YASARA (60), which merged PDB files 5UIW, 5T1A, 5LWE, and 4RWS. The single merged structure was energy minimized with a pH-based pka setting of 7.4 within a phosphatidyl-ethanolamine (PEA) lipid membrane and 0.997g/mL water equilibrated across the membrane using YASARA md_runmembrane macro. Molecular dynamic simulations (mds) were run for the membrane-embedded CCR5 with 14,206 explicit water molecules, 48 Cl, and 33 Na giving a compiled 67,402 atoms for 300 nanoseconds (ns) using the AMBER14 force field (61), and atomic positions collection every 100 picoseconds for analysis. The analysis was performed using YASARA macros md_analyze and md_analyzeres (yasara.org/macros.htm), using a correlation cutoff for each amino acid of >0.9 in dynamic cross-correlation matrix (DCCM) calculations. All the mds trajectory and analysis files can be found at https://doi.org/10.6084/m9.figshare.15134979, allowing for a full reanalysis as needed.

All CCR5 missense and loss-of-function (LoF) variants were extracted from gnomADv2.1 nonTOPmed (62), COSMIC (63), Bravo for TOPmed variants (64), and ClinVar (65) on 11/29/2018. CCR5 missense variants were extracted from Geno2MP (66) on 6/15/2021. All missense and LoF variants were compiled, and each unique change was assessed with PolyPhen2 (67), Provean (68), SIFT (69), and Align-GVGD (70), where the variant was scored 1 for damaging equivalent predictions of each tool. A variant impact score was calculated by adding the functional prediction scores with our codon selection score (max of 2) and multiplying that by the functional conservation score, our linear motif conservation score, and the total allele observations for the variant from gnomAD, TOPmed, ClinVar, COSMIC, and Geno2MP. The top five highest impact scores had the Geno2MP phenotypes extracted on 6/15/2021.

### Public Dataset Generation

The 3D model of CCR5 was recorded for molecular videos using python scripted movement within the YASARA molecular modeling tools (60). The video files were uploaded into FigShare and YouTube, with links provided in the results section. The 3D coordinates were saved as a PDB file and loaded into PyMol (https://pymol.org/2/) to generate colored files for 3D printing, saving the files in VRML format and submitted to FigShare or Shapeways. Delta32 variant allele frequency was extracted from gnomADv2.1 (62). The CCR5 website was built using WordPress.

### RNA Expression Analysis

The genome browser images and all GWAS variants near *CCR5* were extracted from the UCSC genome browser (71) on 9/6/2021. *CCR5* eQTLs were extracted from GTEx version 8 (72) on 9/6/2021. Open Targets Genetics (73) was used for the understanding of GWAS and pheWAS associations. Samples from our previous RNAseq work and details of methods used can be found in the three publications for MODS, RSV, or COVID-19 (74–76). All PAXgene tube blood RNAseq datasets within the NCBI SRA were downloaded with the SRA toolkit (https://trace.ncbi.nlm.nih.gov/Traces/sra/sra.cgi?view=software) and processed for abundance using Salmon_0.14.1 (77) and the Gencode38 transcriptome (78). Microglia datasets were extracted from BioProjects PRJNA649597, PRJNA662330, PRJNA665286, PRJNA667596, PRJNA688478, PRJNA689841, PRJNA387182, PRJNA483247 and the blood RNAseq datasets from BioProjects PRJEB14743, PRJEB20731, PRJEB23048, PRJEB27958, PRJEB27965, PRJEB33892, PRJEB36928, PRJEB41073, PRJEB44660, PRJNA201433, PRJNA230906, PRJNA232593, PRJNA251404, PRJNA277352, PRJNA305001, PRJNA315611, PRJNA327986, PRJNA329148, PRJNA352062, PRJNA354367, PRJNA357628, PRJNA358580, PRJNA369684, PRJNA378794, PRJNA380820, PRJNA384259, PRJNA390289, PRJNA397222, PRJNA398240, PRJNA401870, PRJNA427575, PRJNA437114, PRJNA454694, PRJNA476781, PRJNA493832, PRJNA494155, PRJNA504827, PRJNA511891, PRJNA526259, PRJNA526839, PRJNA533086, PRJNA552286, PRJNA562305, PRJNA588242, PRJNA591657, PRJNA600846, PRJNA601661, PRJNA607120, PRJNA630674, PRJNA632871, PRJNA634938, PRJNA638653, PRJNA639278, PRJNA647880, PRJNA664368, PRJNA679264, PRJNA679331, PRJNA680771, PRJNA683803, PRJNA686397, PRJNA702558, PRJNA728070 in addition to our groups studies on MODS, RSV, and COVID-19 (74–76). All Gencode38 mapped reads for these samples can be found at https://doi.org/10.6084/m9.figshare.16658449.v1. To calculate CCR5 delta32 read frequency we created a fasta file containing all isoforms of CCR5 and several additional paralog isoforms (https://doi.org/10.6084/m9.figshare.16649830.v1) that was indexed and assessed using Salmon, where the percent of reads containing delta32 were compared to the reads without the variant to calculate abundances for the variant from RNAseq datasets.

## Results

### CCR5 Evolutionary Insights

**Figure 1:**
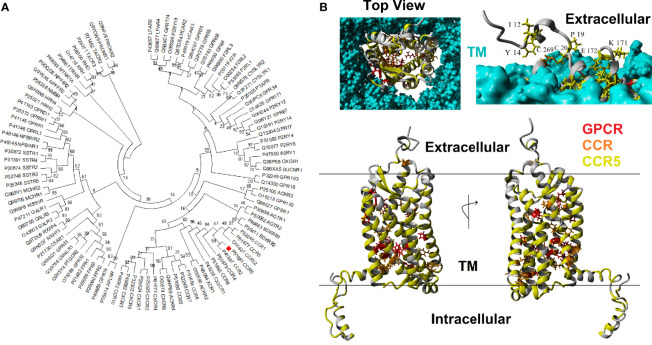
Human CCR5 paralogs. **(A)** Phylogenetic tree of the top 100 human paralogs for CCR5 protein. Values at each node represent the percent of trees that cluster out of 500 bootstrap analyses. CCR5 is marked with a red box.**(B)** Model of human CCR5 with conservation colored. The transmembrane is colored cyan, amino acids conserved >90% in 100 GPCRs in red, conserved >90% in 16 most related CCR5 paralogs in orange, and conserved >90% in CCR5 vertebrate orthologs in yellow. The top left shows the view of CCR5 from extracellular space looking into the GPCR. The top right shows the side view with the transmembrane to visualize exposed and conserved residues, which are labeled. The bottom shows a 180° rotation of side view of CCR5.

CCR5 is a member of the GPCR superfamily. A BLAST analysis of the human CCR5 against other human protein sequences revealed the top 100 hits have E-values less than 5.23E-13, and percent identify greater than 22%. Phylogenetic reconstruction of these 100 GPCR human proteins shows that CCR5 clusters next to CCR2 and near CCR1, CCR3, CCRL2, CCR4, CCR8, CX3CR1, XCR1, and ACKR2 (**Figure 1A**). Using these 100 GPCR sequences, the percent of amino acids the same as CCR5 was calculated for each of the human CCR5 amino acids, where 12 amino acids (3.4%) are conserved >90%. In addition, the top 16 BLAST hits were also assessed for conservation with CCR5, where 26 amino acids (7.4%) are conserved >90%. A total of 98 vertebrate orthologs of *CCR5* were assessed for codon selection, linear motifs, amino acid conservation, and functional amino acid conservation. The alligator CCR5 represents the most divergent sequence within CCR5 orthologs with 54% conservation of amino acids with human. A total of 186 amino acids (52.8%) are conserved >90% in CCR5 orthologs. These conserved amino acids at the GPCR, top 16, and CCR5 ortholog levels mapped onto a model of the CCR5 structure reveal a broad GPCR conservation in the core, 16 most similar conservation in several clusters, and broad CCR5 conservation of the transmembrane, intracellular, and extracellular residues (**Figure 1B**). The fact that other chemokine receptors show a lack of conservation at the ligand-binding interface challenges the notion that they could potentially compensate for CCR5 loss, in agreement with Ellwanger et al. (79).

### CCR5 Amino Acid Knowledgebase

The conservation data from above was compiled with molecular dynamic simulation (mds) data, UniProt insights, and known genomic variants for each of the human CCR5 amino acids to make a CCR5 amino acid knowledgebase (https://doi.org/10.6084/m9.figshare.16619974). The mds were generated by embedding the CCR5 protein of amino acids 6-352 into a PEA membrane, equilibrating water on the intra and extracellular portions, and simulating the protein movement for 300 nanoseconds. The mds tools use physics approximations of atomic movement at the femtosecond time scale, allowing users to determine the chemical environment around each amino acid of the modeled structure, providing information on the stability of movement, secondary structure, and how each amino acid correlates with every other amino acid. By recording the trajectory of amino acid movement using root-mean-squared deviation (RMSD) of the carbon alpha position, we know that the protein reaches an equilibrium of movement around ten nanoseconds of simulation, allowing for us to capture hundreds of nanoseconds of stabilized movement. The seven transmembrane helices of the GPCR structure all have a stable, low movement as reflected by a root-mean-square fluctuation (RMSF) below 4Å. The N- and C-termini both have high levels of RMSF, >10Å, reflective of decreased stability of the structure. A total of 15.0% of the amino acids are predicted in the initial structure to have coiled structure, 76.9% helical, 2.9% beta-sheet, and 5.2% as turns. A total of 70% of the amino acids have one or more amino acids that correlate with their movement greater than 0.9 based on dynamics cross-correlation matrix calculations. A total of eight amino acids have 10 or more amino acids in correlation >0.9 (10 = 54,55,69,149; 11 = 52,66,67; 13 = 70). These calculation values were included in the supplemental file’s main amino acid knowledgebase matrix (https://doi.org/10.6084/m9.figshare.16619974).

Genomic missense variant extraction from gnomAD (population variants useful for allele frequency insights), TOPmed (population variants), ClinVar (disease-associated variants), COSMIC (somatic cancer variants), and Geno2MP (disease-associated variants with correlated phenotype) identified 403 unique variants for CCR5. Based on gnomAD allele frequencies, the average variant was found in 0.0079% of the population, with only a single missense variant (L55Q) found in more than 1% of individuals. Of the 403 variants, they fell on amino acids with an average of 89.6% conservation in CCR5 orthologs with 39% of variants with a conservation >99%. A total of 27% of the variants were predicted probably damaging by PolyPhen2, 54% deleterious by Provean, 60% damaging by SIFT, and 12% as class C55/C65 by Align-GVGD. Only 9% of these variants were predicted bad outcomes in all four tools. To prioritize variant assessments, we used a combined variant impact calculation with an average score of 89,761. The top ten variants were L55Q, R223Q, A73V, V131F, S63C, T288A, L121R, G106R, V46M, R60S. Finally, we added into the amino acid knowledgebase the UniProt extracted data for topology (extracellular, transmembrane helices, or intracellular), posttranslational modification (PTMs: sulfotyrosine, O-linked GalNAc, disulfide bonds, S-palmitoyl, phosphorylation), and known experimental mutagenesis/natural variant insights on ligand binding and protein expression/size. The number of variants and the top impact score for variants were brought into the compiled amino acid matrix with all other datasets, allowing for multidimensional data insights for each variant, for example, the top ten variants and the PTMs (**Table 1**).

**Table 1:** Top functional amino acids of CCR5 from amino acid knowledgebase.

AA	Codon	AA	Inclusion	CCR5 Conserved (%)	GPCR Conserved	Top 16 GPCR (E<1E-50)	Secondary Structure	RMSF (Å)	mds DCCM >0.9
3	TAT	Y	PTM	82.65	2.02	0.00	–	–	–
6	TCA	S	PTM	78.57	14.14	13.33	C	12.161	2
7	AGT	S	PTM	80.61	27.27	6.67	C	9.246	2
10	TAT	Y	PTM	80.61	4.04	13.33	T	4.615	1
14	TAT	Y	PTM	98.98	6.06	33.33	T	3.63	0
15	TAT	Y	PTM	19.39	4.04	20.00	T	4.047	1
16	ACA	T	PTM	27.55	3.03	6.67	C	2.959	1
17	TCG	S	PTM	69.39	7.07	33.33	C	3.384	0
20	TGC	C	PTM	98.97	11.11	53.33	C	1.325	0
46	GTG	V	Top 10	97.96	19.19	40.00	H	1.786	4
55	CTG	L	Top 10	100.00	23.23	60.00	H	1.738	10
60	AGG	R	Top 10	100.00	25.25	33.33	C	2.658	8
63	AGC	S	Top 10	94.90	21.21	46.67	C	1.85	8
73	GCC	A	Top 10	97.96	82.83	80.00	H	1.229	6
101	TGT	C	PTM	100.00	100.00	100.00	H	1.202	7
106	GGG	G	Top 10	91.75	13.13	46.67	H	1.277	4
121	CTC	L	Top 10	100.00	13.13	40.00	H	1.262	5
131	GTC	V	Top 10	100.00	52.53	80.00	T	2.255	0
178	TGC	C	PTM	100.00	10.10	53.33	E	1.055	1
223	CGG	R	Top 10	89.80	20.20	40.00	C	3.459	0
269	TGC	C	PTM	98.97	47.47	93.33	H	1.181	2
288	ACG	T	Top 10	88.78	9.09	26.67	H	1.222	1
321	TGC	C	PTM	89.69	2.02	13.33	C	3.514	0
323	TGC	C	PTM	26.80	0.00	0.00	C	5.452	4
324	TGT	C	PTM	97.94	2.02	13.33	C	6.36	5
336	AGC	S	PTM	83.67	13.13	40.00	C	9.765	4
337	TCA	S	PTM	100.00	22.22	46.67	C	9.244	3
342	TCC	S	PTM	100.00	27.27	40.00	T	7.834	1
349	TCT	S	PTM	98.98	13.13	26.67	C	4.292	1

Sites were included based on known posttranslational modifications (PTMs) or for being a top 10 scoring variant.

### Launch of Public Tools for CCR5 Education

**Figure 2:**
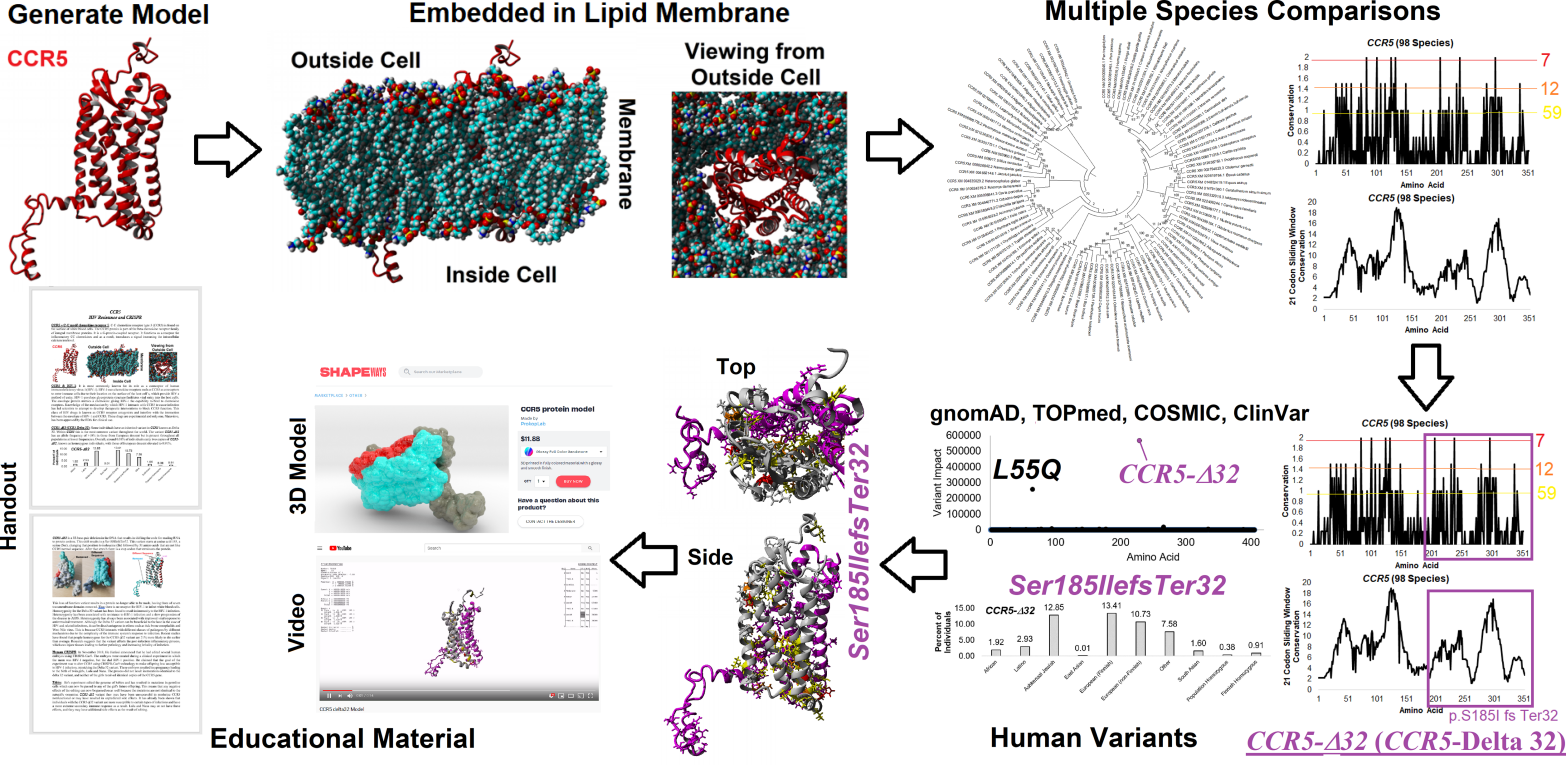
Amino acid knowledgebase of CCR5 used for educational insights. CODE students generated protein models, which were embedded into a lipid membrane and run for molecular dynamics simulations. These values were combined with multiple species analysis of CCR5 evolution and genomic variant extractions from gnomAD, TOPmed, COSMIC, and ClinVar. This amino acid knowledgebase was then used to assess the delta 32 variant and generate various educational handouts, videos, and 3D printed models.

Our CODE students and faculty integrated our amino acid knowledgebase into additional tools and resources for the education of CCR5 protein structural insights, gene sequences throughout many species, and human variants in large databases (**Figure 2**). Many of the tools developed focus on the CCR5 delta32 variant (Ser185IlefsTer32). From the 3D models, we have generated a video of CCR5 and delta32 that brings to light the extreme mutation that results in 0% protein function from the allele. A long ~2 min video is available as a MPG file on FigShare (https://doi.org/10.6084/m9.figshare.16628905) and a video file on YouTube (https://youtu.be/74w2N51tSOg). The video shows the model of CCR5 embedded into a lipid membrane, rotating around all axes with the location of critical conserved amino acids and the delta32 variant. A shortened 14-second video of only delta32 is available as a MPG file (https://doi.org/10.6084/m9.figshare.16628956). A one-minute video of the movement of residues from the mds trajectory is available as an MPG file (https://doi.org/10.6084/m9.figshare.16628854) and a YouTube link (https://youtu.be/WaoPfQXA8Pg). To facilitate a hands-on interface with CCR5, we have created a 3D printing model of CCR5 and delta32 available as a VRML file (https://doi.org/10.6084/m9.figshare.16628962) and as large (https://www.shapeways.com/product/8XEK5N2EF/ccr5-large?optionId=147670860&li=shops), or small (https://www.shapeways.com/product/VNX7TE26B/ccr5-protein-model?optionId=127830241&li=shops) sized print that can be ordered from Shapeways for delivery. The small model provides a low-cost option, which our group has made into jewelry or keychains for distribution at genomic educational events. The large print works well for classrooms for students to hold and explore.

To make access to these files easier, we have created a web resource page (https://prokoplab.com/ccr5-and-hiv/). From this site, anyone can obtain the video of CCR5, sequence data, insights on delta32, and a handout used in classrooms. In addition, visitors can order 3D printed models of CCR5 with the delta32 location marked in red. The two-sided handout (https://doi.org/10.6084/m9.figshare.16628815) walks students through the biological role of CCR5, how many sites on the protein are conserved throughout evolution, and details of delta32. This handout pairs well with the large and small 3D prints of the protein. All material is provided to the public for free except the 3D printed models, offered at production cost with no markup. The generation of this material by students and faculty from the CODE program represents an exciting new potential framework in the genomic era that can be expanded to many additional variants and proteins moving forward.

### CCR5 Expression Insights

**Figure 3:**
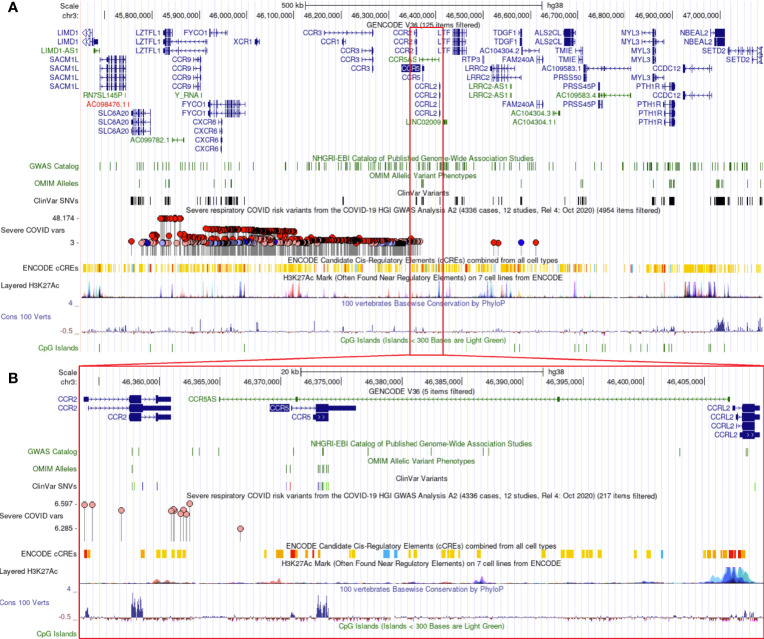
Genomic architecture around CCR5. The genome browser view (hg38) of around one million bases near *CCR5* **(A)** or zoomed into around 40,000 bases **(B)**. Tracks shown include the Gencode transcripts, known variants from GWAS/OMIM/ClinVar, the high-risk COVID-19 loci (higher dots are the strongest signal), various gene regulation insights (ENCODE cCTEs, H3K27Ac, CpG Islands), and evolutionary conservation (Cons 100 Verts).

Additional insights about *CCR5* are available through public datasets of expression and noncoding variants, broadening the insights and knowledge to challenge genomic reductionism. One effective way to combat genetic reductionism is to demonstrate that the expression of CCR5 is not only subject to variation in the gene but is also highly dependent on the molecular context, such as genes proximate to CCR5, epigenetic factors, as well as the type of cell in which expression occurs. The *CCR5* gene is located on chromosome 3 from bases 46,370,854 to 46,376,206 (based on hg38 annotation). Near *CCR5* are multiple cytokine receptor genes and many known genomic associations from genome-wide association studies (GWAS), including the strongest locus for severe COVID-19 (**Figure 3A**). This COVID-19 locus (80) has its highest association signal over the *SLC6A20* gene and does not overlap the *CCR5* gene body (**Figure 3B**). Located around 57 kilobases near *CCR5* are associations for multiple immune system-connected phenotypes notable for lymphocytes, monocytes, and macrophages (**Table 2**). Of the variants in this region, several contribute to observed changes in *CCR5* expression based on expression quantitative trait loci (eQTL), with the strongest associations seen in whole blood and lung, which contain large portions of monocytes and macrophages (**Table 3**). It should also be noted that multiple eQTLs were observed in brain tissue. The top SNP for blood *CCR5* expression influence, rs76258812, is also an eQTL for the other paralogs of cytokine receptors near *CCR5*, including *CCR1*, *CCR3*, and *CCR2* (https://genetics.opentargets.org/variant/3_46318831_T_C), making it difficult to determine if the monocyte associated GWAS are from *CCR5* or these other genes. This variant is also found throughout multiple subpopulations with the highest known allele frequency in East Asian ancestry. Contrary, the top two variants for the brain *CCR5* expression, rs9862021, and rs140177427, are rarer. rs9862021 is found highest in 10% of African ancestry and has no trait associations. rs140177427 is found highest in 10% of Finnish ancestry and is associated with various lymphocyte and macrophage phenotypes (https://genetics.opentargets.org/variant/3_47234712_G_A).

**Table 2:** Traits associated with variants within the *CCR5* gene region (chr3:46,353,419-46,409,888, hg38) of **Figure 3B**.

Study ID	Trait	P-value	Beta	Publication
GCST004433	Macrophage inflammatory protein 1b levels	7.57E-115	0.4985	PMID:27989323
GCST90002340	Monocyte count	1.11E-75	0.035585	PMID:32888493
NEALE2_30190_raw	Monocyte percentage	4.35951E-50	0.0963857	UKB Neale v2
GCST004609	Monocyte percentage of white cells	1.051E-33	0.04394357	PMID:27863252
NEALE2_6149_1	Mouth ulcers | mouth/teeth dental problems	4.03372E-32	0.093964676	UKB Neale v2
GCST004608	Granulocyte percentage of myeloid white cells	2.358E-25	-0.03789086	PMID:27863252
NEALE2_30130_raw	Monocyte count	1.90975E-22	0.00516017	UKB Neale v2
GCST004625	Monocyte count	5.757E-22	0.03506334	PMID:27863252
GCST004438	Monocyte chemoattractant protein-1 levels	1.05E-19	0.2902	PMID:27989323
GCST90002292	Basophil count	4.85E-17	-0.018406	PMID:32888493
GCST90002316	Lymphocyte counts	4.44E-14	0.021045	PMID:32888493
NEALE2_30300_raw	High light scatter reticulocyte count	7.77011E-14	0.000252296	UKB Neale v2
NEALE2_30180_raw	Lymphocyte percentage	2.13306E-11	0.159232	UKB Neale v2
NEALE2_30120_raw	Lymphocyte count	2.24757E-10	0.0233973	UKB Neale v2
NEALE2_30290_raw	High light scatter reticulocyte percentage	3.2664E-10	0.00704487	UKB Neale v2
GCST003045	Ulcerative colitis [EA]	1.3229E-08	0.0757873	PMID:26192919
NEALE2_6149_100	None of the above | mouth/teeth dental problems	2.22533E-08	-0.027486511	UKB Neale v2
NEALE2_30150	Eosinophill count	2.81799E-08	0.00995217	UKB Neale v2
NEALE2_30260_raw	Mean reticulocyte volume	7.16666E-08	-0.137991	UKB Neale v2
NEALE2_30250_raw	Reticulocyte count	8.74646E-08	0.000690186	UKB Neale v2

**Table 3:** Top eQTL for CCR5 expression.

Tissue	SNPs	Lowest P-Value	NES	rsID
Whole Blood	206	1.1E-09	-0.24	rs76258812
Lung	123	3.7E-06	-0.14	rs9110
Brain - Caudate (basal ganglia)	10	1.3E-05	1.1	rs9862021
Brain - Cortex	2	1.9E-05	-1.6	rs140177427
Skin - Sun Exposed (Lower leg)	3	1.9E-05	0.17	rs1388604
Esophagus - Mucosa	47	2.1E-05	-0.17	rs202207288
Esophagus - Muscularis	26	2.4E-05	0.16	rs2133660
Colon - Sigmoid	114	2.7E-05	-0.39	rs6765904
Skin - Not Sun Exposed (Suprapubic)	51	2.8E-05	-0.18	rs9872946
Nerve - Tibial	4	1.0E-04	-0.71	rs80257961

**Figure 4:**
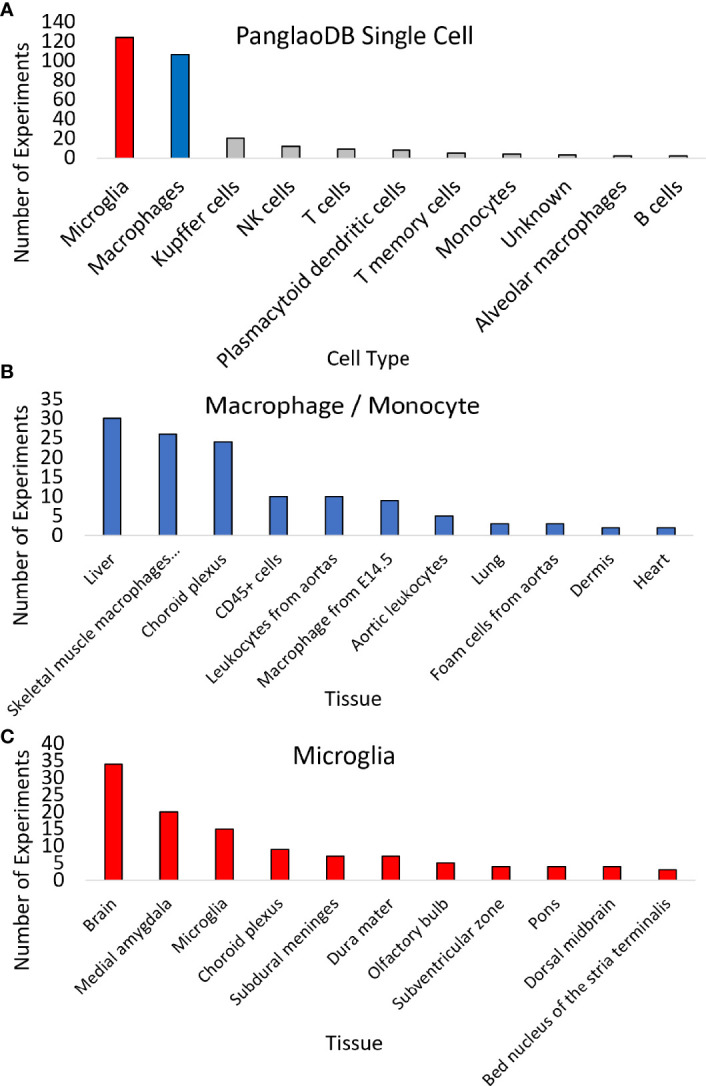
Expression of CCR5 in 1,063 mouse single-cell datasets. **(A)** PanglaoDB analysis of experiments that CCR5 was detected in various cells based on single-cell analysis. **(B, C)** The number of times sample types showed CCR5 expression for macrophages/monocytes **(B)** or microglia **(C)**.

Expression across broad tissues of the human protein atlas (HPA) (29) and GTEx (72) show higher levels in immune tissues such as spleen, tonsils, appendix, and lymph nodes and tissues associated with immune cells like blood, lung, and intestine. Further dissection of cell types within HPA shows high *CCR5* expression in T-cell levels in blood and macrophages in peripheral tissues. Therefore, we utilized a large single-cell RNAseq repository, PanglaoDB (81), to identify further cell types of importance for *CCR5* expression, identifying a large number of microglia and macrophage identified cell experiments (**Figure 4A**). The macrophage and monocyte annotations come from broad tissues, including the liver, vessels, lung, and heart (**Figure 4B**). The microglia annotations come from whole-brain isolates and specific brain regions (**Figure 4C**). It should be noted that all of the microglia insights come from isolates of mouse brains, where there are no human brain single-cell RNAseq datasets integrated into PanglaoDB. In the mouse, the knock-out of *CCR5* is associated with multiple neurological phenotypes, including microglial alterations and abnormal spatial learning (**Table 4**).

**Table 4:** Mouse knock-out phenotypes.

Phenotype	Publication	Neurological
abnormal astrocyte physiology	(82)	Yes
abnormal glial cell physiology	(82)	Yes
abnormal long term spatial reference memory	(82)	Yes
abnormal nervous system physiology	(83)	Yes
abnormal spatial learning	(82)	Yes
decreased microglial cell activation	(82)	Yes
abnormal CD4-positive, alpha beta T cell morphology	(84)	
abnormal CD8-positive, alpha beta T cell morphology	(84)	
abnormal cytokine level	(84)	
abnormal hepatocyte physiology	(85)	
abnormal Ito cell morphology	(86)	
abnormal Kupffer cell morphology	(86)	
abnormal locomotor behavior	(84)	
abnormal NK T cell physiology	(85)	
decreased NK cell number	(87)	
decreased susceptibility to induced colitis	(84)	
decreased susceptibility to Retroviridae infection	(83)	
impaired macrophage chemotaxis	(88)	
increased NK T cell number	(84)	
increased susceptibility to fungal infection	(88)	
liver failure	(85)	

### CCR5 Role in Brain Development

**Figure 5:**
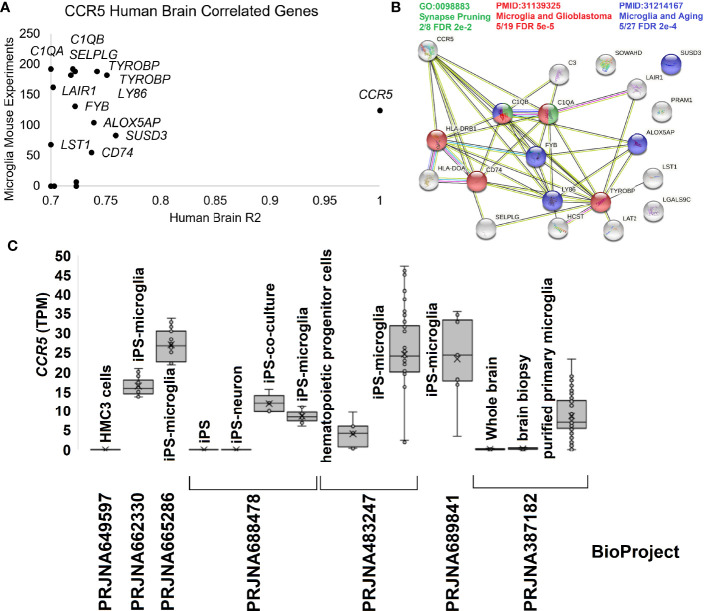
Human brain CCR5 and microglia. **(A)** The correlation of genes to CCR5 from the Allen Brain Atlas Human Brain microarray data for 500 samples relative to their expression in mouse microglia single-cell experiments. The x-axis shows the genes R^2^ from microarray relative to CCR5 expression, while the y-axis shows the number of experiments the gene is detected in mouse single-cell datasets for microglia. **(B)** STRING protein network for genes in panel **(A)** showing the enrichment of synapse pruning (green), microglia/glioblastoma (red), and microglia/aging (blue) genes. **(C)** Expression of *CCR5* in transcripts per million (TPM) from seven BioProjects of human microglia cells or related experiments.

To further dissect the brain and microglia insights into human biology, we integrated several additional datasets. The human brain microarray of the Allen Brain atlas suggests 16 genes that correlate >0.7 in expression with *CCR5* in 500 human brain samples. Most are also highly expressed in the mouse single-cell datasets of PanglaoDB for microglia (**Figure 5A**). Most of these genes code for proteins that are known to interact and enrich synapse pruning and microglia phenotypes (**Figure 5B**). To further show the expression of *CCR5* in isolated microglia, we pulled all human RNAseq paired-end datasets from the NCBI SRA mentioning “microglia.” We annotated them relative to the Gencode38 transcriptome using a quasi-based alignment strategy (**Figure 5C**). All Gencode38 mapping data for each sample can be found at https://doi.org/10.6084/m9.figshare.16649842. The HMC3 cell line (PRJNA649597) that is supposed to mimic microglia cells does not express any *CCR5*. Contrary iPS (induced pluripotent stem cell) derived microglia (PRJNA662330, PRJNA665286, PRJNA688478, PRJNA483247) and purified primary microglia (PRJNA387182) all show expression of *CCR5*.

**Figure 6:**
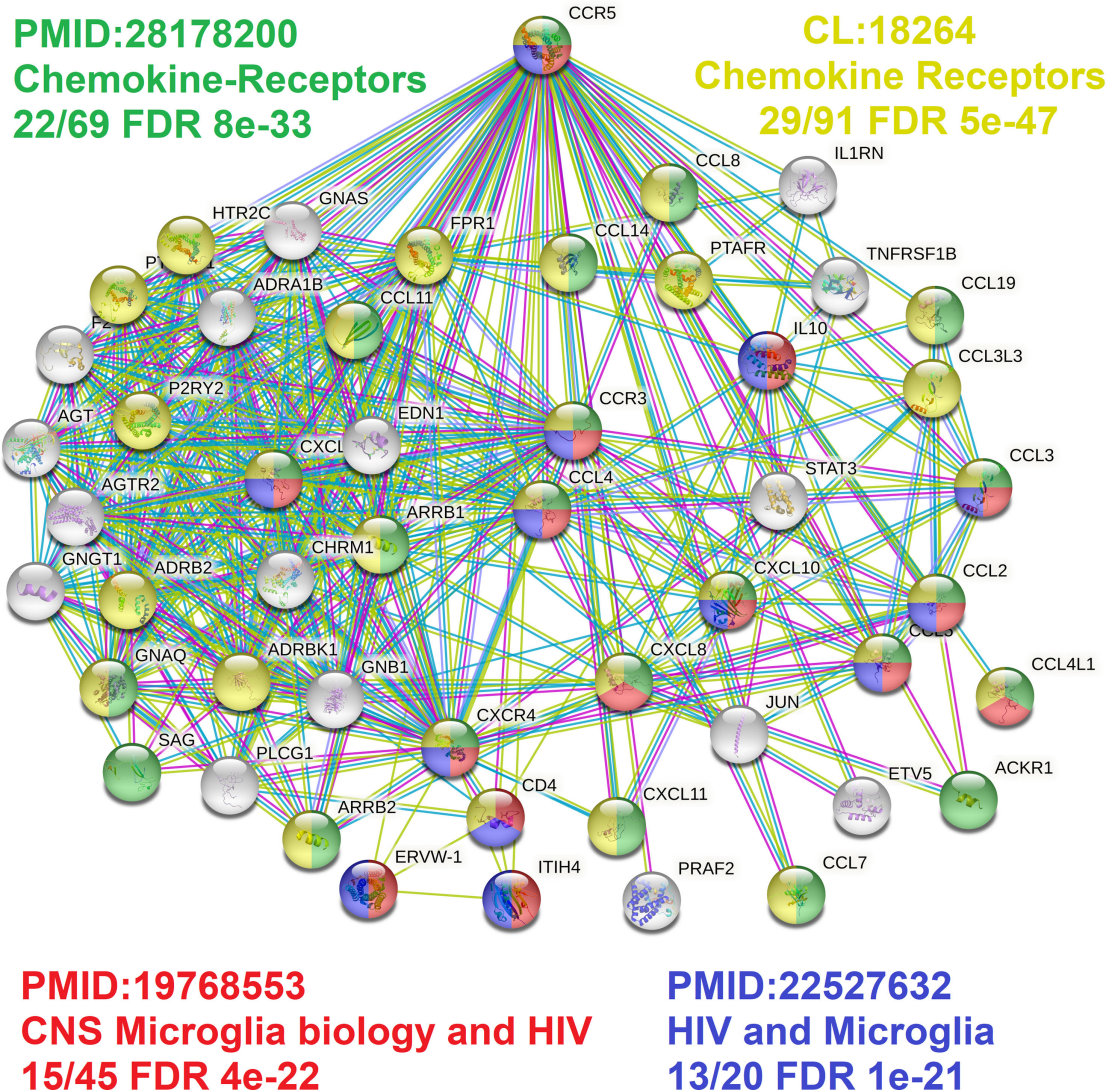
CCR5 protein network highlighting microglia factors. The STRING protein network for the top 50 proteins interacting with CCR5. In green/yellow are various genes annotated to chemokine biology, and in red/blue are genes connected to microglia biology.

Broadening to the more extensive integrated STRING network for CCR5 shows interaction with many chemokines while identifying multiple genes connected to the central nervous system and microglia biology relative to HIV (**Figure 6**) (89, 90). Using our amino acid knowledgebase, we ranked the top five variants (L55Q, R223Q, A73V, V131F, S63C) followed by extracting human phenotypes associated with these changes in the Geno2MP database. A total of 273 affected individuals have a phenotype with one of these top five variants. Of these, 73 affected individuals (27%) are indicated as “Abnormality of the nervous system” in the broad term (**Table 5**). This is the top-ranked phenotype with “Abnormality of the cardiovascular system” at 21% and “Abnormality of the musculature” at 11%. The only neurological phenotype associated with homozygous CCR5 variants was L55Q associated with “Abnormality of brain morphology” with seven additional heterozygous individuals with the variant and individuals with R223Q, A73V, V131F, and S63C also having this annotation. Individuals with all five variants are also identified with “Intellectual disability”. Several variants are associated with “Epileptic encephalopathy”. The L55Q is found in ~1.5% of the population, and therefore association to phenotypes could be random; however, the 21 patients with neurological phenotypes with R223Q (AF=0.005) and 18 with the other three (A73V, S63C, V131F, AF average of 0.0006) suggest an enrichment from random probability. The Geno2MP database contains data for 19,344 individuals with phenotypes, where 458 have one of these five CCR5 variants. Based on allele frequencies, this number is expected to be 384, with an enrichment of 1.2. The V131F has an enrichment of 8.2 in observations relative to expect allele frequency, A73V of 4.3, and S63C of 2.2, suggesting these variants are hyper observed within Geno2MP. Moreover, with the enrichment of neurological phenotypes for individuals with these variants, it seems likely that there is an association with rare variants in CCR5 for neurological phenotypes.

**Table 5:** Neurological phenotypes for the top human CCR5 missense variants from Geno2MP.

Variant	Het	Hom	Phenotype
L55Q	7	1	Abnormality of brain morphology
L55Q	4	0	Epileptic encephalopathy
L55Q	4	0	Abnormality of nervous system physiology
L55Q	3	0	Neurodevelopmental abnormality
R223Q	3	0	Intellectual disability
L55Q	2	0	Microcephaly
L55Q	2	0	Dystonia
L55Q	2	0	Fatigable weakness
R223Q	2	0	Agenesis of corpus callosum
R223Q	2	0	Abnormality of brain morphology
R223Q	2	0	Cerebral cortical atrophy
R223Q	2	0	Abnormality of nervous system morphology
A73V	2	0	Epileptic encephalopathy
S63C	2	0	Intellectual disability
L55Q	1	0	Autism, Intellectual disability
L55Q	1	0	Spastic paraplegia
L55Q	1	0	Abnormality of hindbrain morphology
L55Q	1	0	Seizures
L55Q	1	0	Abnormality of nervous system morphology
L55Q	1	0	Intellectual disability
L55Q	1	0	Global developmental delay, Autism
L55Q	1	0	Global developmental delay
L55Q	1	0	Abnormality of the nervous system
R223Q	1	0	Behavioral abnormality
R223Q	1	0	Seizures
R223Q	1	0	Neurodegeneration
R223Q	1	0	Abnormality of hindbrain morphology
R223Q	1	0	Seizures
R223Q	1	0	Epileptic encephalopathy
R223Q	1	0	Abnormality of nervous system physiology
R223Q	1	0	Intellectual disability
R223Q	1	0	Intellectual disability
R223Q	1	0	Global developmental delay
A73V	1	0	Intellectual disability
A73V	1	0	Agenesis of corpus callosum
A73V	1	0	Abnormality of hindbrain morphology
A73V	1	0	Abnormality of brain morphology
A73V	1	0	Intellectual disability
V131F	1	0	Abnormality of hindbrain morphology
V131F	1	0	Abnormality of brain morphology
V131F	1	0	Fatigable weakness
V131F	1	0	Abnormality of nervous system physiology
S63C	1	0	Spastic paraplegia
S63C	1	0	Microcephaly
S63C	1	0	Abnormality of brain morphology
S63C	1	0	Seizures
S63C	1	0	Abnormality of movement

The Het is the number of heterozygous individuals with the variant and the annotated phenotype and Hom are homozygous individuals with phenotype.

### CCR5 in Human Blood Samples

**Figure 7:**
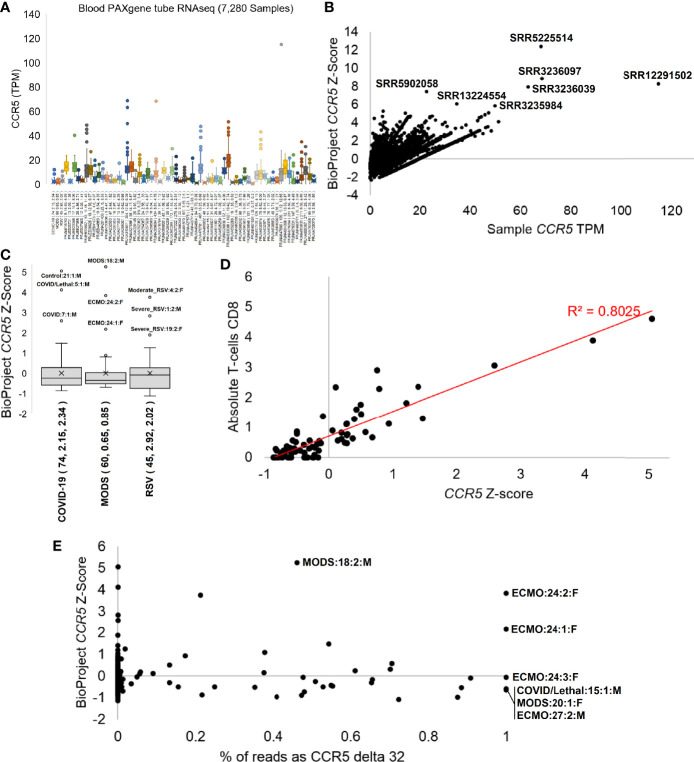
CCR5 expression in blood PAXgene tube RNAseq. **(A)** Box and whisker plots for the expression (TPM) of *CCR5* from various NCBI BioProjects that were generated by Illumina paired-end RNA-Seq from human blood collected PAXgene tube samples. Listed next to each BioProject code is the number of samples, average *CCR5* expression, and standard deviation of CCR5 expression. **(B)** The TPM expression for each sample (x-axis) of panel **(A)** relative to the BioProject normalized Z-score (y-axis). The top seven samples based on Z-score are labeled. **(C)** The Z-scores for our three pilot precision transcriptome datasets, with outlier samples labeled. **(D)** The COVID-19 study analysis of *CCR5* normalized expression relative to CIBERSORTx absolute CD8 T-cell values. **(E)** The percent of transcripts containing the delta 32 variant relative to wild type (x-axis) for samples of panel **(C)** relative to the BioProject normalized Z-Score. All homozygous samples for delta 32 are labeled as is the heterozygous sample with the highest study Z-score.

The phenotypes seen in the Geno2MP data suggested a broader analysis of CCR5 in disease pathologies. With the robust expression of *CCR5* in blood and our history in studying the blood of immune challenged individuals, we selected to process *CCR5* biology for blood-based RNAseq of 7,280 samples from 62 different BioProjects and three of our studies where we have patient-to-transcriptome insights (74–76) (**Figure 7**). All these samples were blood collected into RNA PAXgene tubes to standardize sample collection. Expression of *CCR5* is highly variable across BioProjects (**Figure 7A**), suggesting that the TPM data is influenced by the RNA isolation or sequencing technique (polyA vs. total RNAseq). Therefore, we normalized the TPM data with a BioProject Z-score, identifying several samples with elevated CCR5 expression (**Figure 7B**). The highest Z-score was observed in SRR5225514 (12.4), a 17-year-old (yo) female control sample, yet very little is deposited into the SRA about this individual. This can also be said for SRR3236097 (8.8, 17 yo male control), SRR12291502 (8.3, male sepsis case), SRR3236039 (8.0, 17 yo male TB patient), SRR5902058 (7.4, male malaria vaccinated individual), SRR13224554 (6.1, 41 yo HIV-infected patient), and SRR3235984 (5.9, 14 yo female TB patient).

Therefore, we focused analysis on three cohorts our team collected blood PAXgene tube RNAseq in hospitalized patients with MODS, RSV, or COVID-19 in an age range from weeks of life to elderly. The adult hospitalized COVID-19 cohort shows highest *CCR5* expression in the male Hispanic control patient 21 age 50-59 (**Figure 7C**), who had the highest interferon response, was noted to have a unique transcriptome, was the furthest outlier of the control samples, had markers of multiple organ damage, and generally seemed to be a highly divergent sample from the cohort (74). The second highest was a 50-59 yo European ancestry male who had a lethal case of COVID-19 marked by a SAPSII score of 61, suggesting multiple organ complications, had a robust interferon response, elevated cytokine expression profile, the highest cell markers of peripheral monocytes, and a weak clonal expansion of the immune repertoire. The third outlier of the study was a 40-49 yo European ancestry male with a SAPSII score of 30, a high activation of mitotic cell cycle control genes, an elevation of interleukin-7 and histone genes, and detectable reads in the blood related to *Paraburkholderia* and *Streptomyces tsukubensis*. None of the samples were identified as outliers of *CCR5* expression on the low end, but samples with *CCR5* expression <0.8 standard deviations were all noted in COVID-19 hospitalized patients and not controls. The depth of sequencing for this COVID-19 study allowed for various gene panels, immune repertoire, and foreign RNA mapping for all samples, which were correlated to the *CCR5* values. The CIBERSORTx absolute values for CD8 T-cells were highly correlated (R^2 =^ 0.8) to *CCR5* expression (**Figure 7D**).

Further evaluation of our pediatric MODS and infant RSV cohorts (**Figure 7C**) reveals diseased samples as outliers of *CCR5* expression. Sample 24, female 16 yo with European ancestry, within the MODS study had two separate measurements with high *CCR5* and an *Epstein Barr* virus (EBV) infection that required ECMO and was also identified as having the highest levels of CIBERSORTx annotated CD8 T-cells (75). Sample 18, a male who required ventilation, had the highest levels of *CCR5* within the MODS cohort and was noted to have elevated genes for interferon response, had a clinical *Serratia marcescens* infection, and was coronavirus positive. The lowest *CCR5* levels in the MODS cohort were observed in sepsis patients, one of which (patient 27) was lethal. The RSV study’s top three outliers for CCR5 expression were all hospitalized RSV cases (patient 1, 4, 19) (76). Patient 1 had high levels of *Agromyces aureus* and *Caulobacter vibrioides* like reads in the blood in addition to clinically confirmed RSV, while also having multiple levels of elevated RNA associated with lung hyperinflammation. Patient 4 had elevated reads belonging to type-1 Alveolar cell and an elevation of viral defense response genes relative to the cohort. All three samples with a z-score below -1 were from hospitalized RSV samples. These three cohorts suggest that *CCR5* levels, either high or low, often are found in sick patients and rarely seen in healthy controls within our cohorts.

To probe whether RNAseq samples contained the *CCR5* delta32 variant, we developed a novel Salmon-based indexing file containing the wild-type *CCR5* transcripts, the delta32 transcripts, and several of the top human paralog transcripts. Mapping samples with delta32 reads over our 179 in-house RNAseq samples reveals 75.4% of the samples to be homozygous wild type (<0.01% delta32 reads), 21.2% heterozygous, and 3.4% homozygous for delta32 (**Figure 7E**). The heterozygous samples were highly variable for the % of reads with delta32 and included the MODS sample 18, with the highest overall *CCR5* z-score of the three studies. Surprisingly, all three samples of the ECMO patient 24 showed homozygous *CCR5* delta32, where an additional two-year follow-up of the patient also showed homozygous *CCR5* (data not shown as the z-score cannot be calculated as it was a single sample RNAseq). This patient was the focus of our 2020 MODS paper (75), where we discovered the 16 yo patient to have Hemophagocytic lymphohistiocytosis (HLH) likely driven by a dominant-negative splicing variant in RNASEH2B that is activated by the EBV suppression of nonsense-mediated decay. As this patient is an N=1 case, it is interesting to note the severe, nearly lethal phenotype of this patient and to be *CCR5* delta 32 homozygous, suggesting that *CCR5* complete inhibition does not remove HLH risks as others have proposed for COVID-19 (91). It is also interesting that as the EBV infection cleared in the patient, the *CCR5* levels, even when delta32 homozygous, decreased in sample 3. The other three samples where we observed *CCR5* homozygous delta32 were in severe outcomes, including patient 15 who had lethal COVID-19, the MODS patient 20 who had Alveolar rhabdomyosarcoma and human betaherpesvirus 7, and the MODS patient 27 who required ECMO and passed away. Our cohort of 98 unique individuals from these three studies had only 13 lethal cases, of which two were delta32 positive (15%). This trend demands further evaluation of delta32 status in sepsis and critical care patients.

## Discussion

Creating modification in genomic backgrounds that are not commonly present with the variant yields unknown risks that need to be assessed before moving to human editing, particularly *in utero*. The delta32 variant is unique to subpopulations of humans, and there could be unknown variants co-evolved in these populations that modulate the deleterious effects of the delta32 variant. As *CCR5* is found within a region of the genome containing multiple cytokine receptor paralogs and has a large and complex linkage disequilibrium block and eQTLs often overlap the different paralogs, genetic variants can be in linkage disequilibrium to compensate for deleterious outcomes. In other pathologies, these shared variants that regulate counter genes have been shown for cardiovascular biology and present on the Y-chromosome (92, 93). Viewed in the context of evolution by natural selection, selection on one trait has correlated effects and is often constrained if those correlated effects are themselves deleterious (94, 95). Editing genomes is independent of evolutionary selection over time and could result in unintended medical consequences due to genomic backgrounds used in human editing. Bioinformatic studies of susceptibility loci suggest that pleiotropy, the existence of multiple functions for a single gene, far from being the exception, appears to be the rule, not only in the case of CCR5 but also in the case of susceptibility loci for breast cancer, lung cancer, coronary artery disease and other severe diseases (96). Thus, understanding the effects of CCR5 removal in diverse genome backgrounds is critical before introducing the variants in diverse human backgrounds. These studies are very complex, requiring extensive data, experimentation, and a great deal of financial resources and time.

An additional set of ethical, legal, and social implications has been brought to light by the He Jiankui affair, in which CRISPR-Cas9 gene editing was used in an attempt to edit the CCR5 gene of two embryos to the Δ32 variant with the hope of conferring immunity to HIV. The germ line was crossed, and twin baby girls, Nana and Lulu, were born. This universally condemned ethical breach leaped over several ethical safeguards. Before translating germline gene-editing to the fertility clinic, it will be necessary to carefully assess the ethical and legal conditions for its permissibility and move toward finer line-drawing in determining these conditions (97). For example, there is a significant biological and ethical difference between a “correction” of a rare, disease-associated mutation to a widespread non-pathogenic allele as is being investigated for HBB and MYBPC3 in the case of β-thalassemia and hypertrophic cardiomyopathy respectively, and attempted edits to CCR5 with its complex, multiple phenotypic effects and potentially pathogenic off-target effects (97, 98). Germline gene-editing carries with it its own set of ethical issues, most notably extreme uncertainty about the effects on the edited individual, as the effects of edits—whether those intended, errors, or off-target—to the genome early in life ramify unpredictably throughout embryogenesis and later development, as well as concerns about long-term intergenerational effects that are difficult to study and whose risks cannot be easily assessed.

Genetic and genomic reductionism takes many forms, and the terms are variously used in the philosophy of biology (99). The particular form of genetic reductionism under criticism here is the mistaken belief that a given gene stands in a strict one-to-one relationship to a given character state, whereas a one-to-many relationship is the rule (99). With rare exceptions, causal pathways in biology are complex. Yet, in the case of genomics, there is good reason to believe that a reductionistic tendency has been inherited from a long-standing biomedical model (100). Reductionism has played an especially important role in the history of understanding pathogenicity, with the locus classicus being Koch’s postulates, whereby it can be experimentally demonstrated that a single species of microorganism is the cause of a particular disease state (100). Based on the prevalence of “gene-for-X” publications in the genomics literature, the reductionistic biomedical model that underlay Koch’s postulate throughout the expansion of the paradigm of the germ theory of disease appears to have been quite widely adopted in genomics research, despite acknowledgment of the complexity and context-dependence of gene expression, pleiotropy, and other effects that belie reductionism (96, 100). Genetic reductionism is best understood as a research strategy from which much is learned in its failures, which point to additional elements of the overall biological context necessary for fuller understanding (101, 102). In other words, methodological reductionism should be understood as a heuristic device for uncovering ontological complexity (101).

An explicit ethical framework, modeled on informed consent, is necessary to undergird a careful, thoughtful approach to assessing and considering the ethical, legal, and social implications of genomic research in which CODE can play a crucial role. In the same way that it has been urged that students of biology need a thorough understanding of the tools and principles of ethics (103), the public, as stakeholders in assessing the ethical, legal, and social implications of genomic research and genomic medicine, need an understanding of the complexities of molecular biology to the extent that this can be achieved. The importance of providing tools for a broad range of educational encounters to build a more complex and realistic understanding of genomics is necessary if the public is to be consulted on research oversight, policy, and clinical application. As research proceeds in the study of CCR5 and other potential intervention targets for germline gene editing, a complex conversation is unfolding in which scientists, when consulted on the need for ethical boundary policing and a cautious approach to moving from basic to translational research to potential application, are pointing to the need to include the public in deliberations over where to draw the line in gene therapy more broadly and more specifically in interventions and genomic edits that cross the germ line (104). An analogy can be drawn with informed consent, a pillar of biomedical ethics, whereby if the public’s voice is to play a role in providing ethical guidance for genomics research and genomic medicine, it is necessary for the public to be informed. CODE can serve as a platform for providing the sorts of educational tools to be deployed in various settings to improve understanding of the science of genomics.

Complicating research and public understanding, assessing the quality of large public databases has been challenging. During the middle of 2019, a Nature Medicine study brought to light the possibility that delta32 decreases life expectancies, yet the results were retracted due to errors in genotyping. This retraction has had consequences of potential public misinformation, suggesting that delta32 has no negative impact, even though the study only addressed a few associations, and public stories rarely discuss the known risks of delta32 (27), something to which our blood RNA-seq also lends support. The removal of CCR5 is not the only possible solution to developing HIV resistance, with the FDA fast-track work on compounds like Leronlimab to antagonize CCR5 binding by HIV. If these compounds are successful, then genomic modification may be unnecessary, with medications offering the safer approach for the patient. More importantly, all potential treatments need to be considered in the light of all available data, including their role in modulating neurodevelopment and sepsis outcomes. These discussions have diverged from the central question of what CCR5 does within cells, why it is important, and the severity of variants like delta32, which removes multiple transmembrane helices and thus prevents the correct proteins production and cellular localization. Further discussions are critically needed into potential gain-of-function roles of misfolded CCR5 delta32 (105) and our interesting observation of the broad distribution of blood CCR5 delta32 RNA-based allele frequencies in heterozygous individuals and accumulation of RNA in one individual that was homozygous (**Figure 7E**). Thus, the development of educational material for projects like CCR5 is critical for a more robust view of the protein, its variants, and its diverse physiological functions outside of what is emphasized in popular media.

Further complicating the role of CCR5 in human biology is the interplay of cytokine biology with CCL3, CCL4, and CCL5 (primary ligands for CCR5). The role of these chemokines in biology and disease have been well studied, and they are known to play a role in asthma, viral infections, dengue fever, acute kidney disease, and multiple sclerosis, with the oscillation of their role between benefit and harm depending on circumstance and insult (106–110). Although seen as inflammatory mediators, these chemokines are induced by inflammatory cytokine in multiple models, including neural inflammation. Typically treated as a site of immune privilege, resident macrophages, or glial cells within the brain, play an integral role in formulating a proper inflammatory response in a state of infection or injury. Microglia have been shown to secrete the inflammatory cytokines IL-1β and TNF-α, resulting in increased secretion of CCL3 and CCL4 (111), further supporting the role of CCR5 in microglia function and feedback.

Similarly, treatment of human brain endothelial cells with IL-1β, TNF-α, and IFN-γ resulted in a significant increase in the secretion of CCL3, CCL4, and CCL5, likely resulting in an increased capacity for leukocyte extravasation through integrin activation (110, 112). The interplay of cytokines in this paradigm is not monodirectional. CCL5 can skew the host’s CD4 T cell response to pathogen from a TH2 phenotype to a TH1 phenotype, altering cytokine secretion. This modulation results in the release of proinflammatory cytokines such as TNF-α and IFN-γ, further potentiating the skewing (108). The biological complexity of CCR5, its ligands, and the interplay of the immune response as a whole demonstrates the need for a more nuanced discussion of our understanding of the available data.

Microglia, the resident macrophages of the central nervous system, express CCR5 as discussed. Microglia contribute to the blood-brain barrier (BBB) accompanied by a meshwork of endothelial cells, astrocytes, pericytes, and a basement membrane (113). In early development, microglia contribute to the pruning of neurons, with dysregulation of this process known to contribute to autism spectrum disorders (114). The ligands for CCR5 (CCL3, CCL4, and CCL5) display expression profiles from astrocytes, endothelial cells, microglia, and within specific subpopulations of neurons (112, 115). CCR5 expression and activation by its respective ligands promote CCR5+ leukocyte adhesion and transmigration through the blood-brain barrier (116). In response to CNS insults, CCR5 is upregulated, prompting an inflammatory response (117). In stroke, CCR5 knock-out is established to increase the severity of brain injury (118). However, this function of CCR5 in pathological states is either beneficial or detrimental depending on the inciting event. Studies with infectious agents such as *Toxoplasma gondii* (119), Herpes Simplex Virus type 1 (120), and Herpes simplex virus type 2 (121) have shown that knocking out *CCR5* contributes to enhanced infection and increased disease severity, likely due to blunting of the immune response.

On the contrary, malarial infection with cerebral involvement in *CCR5* knock-out mice resulted in neuroprotection by decreasing lymphocyte migration and destruction of the CNS (122). Much like our blood *CCR5* expression analysis, this data suggests a balance of infection to outcomes based on CCR5 in the brain. Despite the prominent immunological role of CCR5, both peripherally and centrally, downregulation of *CCR5* does not reduce immune microglial transmigration in the CNS during pathological insults (123), indicating that there are other alternative pathways able to compensate for a dampened immune response. Constitutive basal levels of *CCR5* expression within the CNS in the absence of pathology indicate ancillary roles for this chemokine receptor outside of the known immune system component-with studies supporting its involvement in neural development and physiological functioning.

The complex role CCR5 plays in the central nervous system beyond the immune system has not been fully fleshed out; however, it has been demonstrated to play a role in fetal CNS development, neuronal differentiation, and neuronal survival. In a study by Westmoreland et al., it was found that *CCR5* was expressed by primate fetal cells with increasing expression from birth to 9 months of age (124). Further studies have shown that CCR5 activation results in neuronal differentiation and neuronal survival during apoptotic states (125, 126). The expression of chemokine receptors, such as CCR5, plays a role in neural progenitor cell migration and neuronal connections (127). Collectively, it appears that CCR5 plays a role in neural cell migration, differentiation, and survival during the postnatal period of CNS development, correlating to the timing of microglial pruning. This conclusion is also supported by the fact that many CNS tumors upregulate CCR5 during their rapid growth, including glioblastoma multiforme (128) and primary CNS lymphoma (129). Further insight into CCR5’s role in the CNS is needed to determine the specific mechanisms at which it is involved outside of the known immune functions.

CODE projects lower the barriers to engaging in bioinformatics research by placing genomic analysis within the grasp of a broad and diverse audience. A typical project path mirrors the approach taken by clinical research analysts, beginning with database research, followed by modeling and simulations, and culminating in data analysis. Students first identify a genetic variant of interest identified through genomic research at HudsonAlpha, Michigan State University, or from a publicly available genome database like ClinVar (human). Students use publicly available databases and relevant software to learn more about the gene containing the variant and its functional product. Students compare the genomic sequence with similar segments from other organisms to study the evolutionary conservation of both DNA and protein. This information helps in making predictions regarding the functional consequence of the DNA variant.

Students next employ molecular modeling to study how variants may alter the protein. This type of molecular visualization provides essential support for reasoning on and formulating hypotheses related to molecular structure. Current software tools such as YASARA, PyMOL, and UCSF Chimera allow students to build a 3-D model of a protein, insert a variant, and visualize whether the variant changes protein folding and structure. Students can then study the DNA variant in a computationally-derived cellular environment, running simulations to predict how the variant-containing protein might behave inside a cell. Much like this study, that molecular level knowledge can be combined with analysis of multiple expression databases to allow the students to identify the cell types and tissues where the variants might alter function, known and genotype-to-phenotype insights. This type of analysis can facilitate understanding of the variant’s effect and even provide insight into interventions to offset potentially damaging impacts. Students document their work and findings of their genetic variant in written reports, poster presentations, and research talks at their schools and scientific conferences and contribute to published research in relevant journals. As a byproduct, the students often generate educational resources such as 3D models, videos, and worksheets that can inform clinical analysis and broader understanding of genetic variants, such as shown here for CCR5.

This report on the impact of studies fueled by academic resources shed light on the need for and potential impact of applying education resources to cases in genomics. In the growing era of precision medicine, the need for tools to define the effects of variants (90) and propagate the information to patients and the public has grown, especially to combat genomic reductionism. CODE provides undergraduate students at diverse institutions with an improved understanding of genetics and developing educational material. As knowledge continues to grow for CCR5, several of the CODE students can continue engaging in molecular-level research, such as simulating the recently solved CCR5 ligand-bound structures (130) and integrating the knowledge into our tools. We have expanded these tools to clinical variants from the HudsonAlpha genomic sequencing projects to help explain complex Variants of Uncertain Significance (VUS), including MED13 (131) and RALA (132) that can be found at prokoplab.com/educational-resources/. At the outbreak of the SARS-CoV-2 pandemic, we also used this same workflow to establish critical and rapid insights on the viral encoded proteins and their interactions with host proteins utilizing CODE as a distributed research network to gain faster insights of proteins (133, 134). Expanding these tools to additional projects and collaboration will open a door for educational information in the needed area of genomics and genomic medicine.

## Data Availability Statement

The datasets presented in this study can be found in online repositories. The names of the repository/repositories and accession number(s) can be found in the article/supplementary material.

## Author Contributions

JB, RS, RO, JM, AE, CS, SC, and JP contributed to the generation of CCR5 amino acid knowledgebase or expression analysis. MM, LB, CLS, DH, and JP contributed to the generation of CCR5 educational material. JB, RS, RO, JM, AE, JZ, NH, BC, SR, CB, and JP contributed to the analysis and interpretation of 3 house RNAseq studies. JH contributed philosophical oversight of genomic reductionism. MM, CLS, SC, BC, SR, CB, and JP supervised the completion of all work. JB, MM, JZ, and JP wrote the manuscript. All authors contributed to the article and approved the submitted version.

## Funding

This work was supported by the Spectrum Health and MSU Alliance Corporation, Spectrum Health Foundation, National Institutes of Health (K01ES025435 to JP), Gerber Foundation, and Michigan State University.

## Conflict of Interest

The authors declare that the research was conducted in the absence of any commercial or financial relationships that could be construed as a potential conflict of interest.

## Publisher’s Note

All claims expressed in this article are solely those of the authors and do not necessarily represent those of their affiliated organizations, or those of the publisher, the editors and the reviewers. Any product that may be evaluated in this article, or claim that may be made by its manufacturer, is not guaranteed or endorsed by the publisher.
